# Emerging Tools and Techniques for Catheter Ablation of Cardiac Arrhythmias: A 2024 Update

**DOI:** 10.19102/icrm.2024.15019

**Published:** 2024-01-15

**Authors:** Arash Aryana, André D’Avila

**Affiliations:** 1 Mercy General Hospital and Dignity Health Heart and Vascular Institute, Sacramento, CA, USA; 2 The Harvard Thorndike Electrophysiology Institute, Beth Israel Deaconess Medical Center, Boston, MA, USA

**Keywords:** Atrial fibrillation, catheter ablation, posterior wall, pulsed-field ablation, ventricular tachycardia

Cardiac electrophysiology has seen steady and remarkable progress largely through the refinement of mapping and ablation tools and techniques, particularly for the catheter ablation of complex arrhythmias such as atrial fibrillation (AF) and scar-mediated ventricular tachycardia (VT). These developments have not only led to greater procedural safety and efficacy but also improved efficiency. Yet, the best may be to come. The ensuing years are going to bring novel energy modalities and disruptive technologies as we further our mechanistic understanding of complex arrhythmias and optimize our ablation strategies. These developments have the potential to significantly transform our field and even drive many types of ablation procedures from the hospital to the ambulatory setting.

## Ablation techniques and strategies for atrial fibrillation

**Figure 1:**
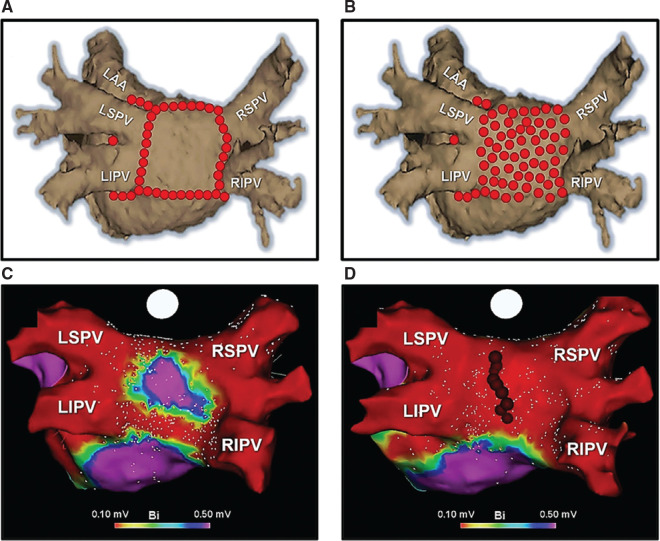
Posterior wall ablation techniques. Shown are the techniques of posterior “box” **(A)** and “direct” posterior wall **(B)** ablation using point-by-point radiofrequency. **C:** When performing posterior wall ablation using the “box” method, in many cases, despite seemingly continuous lesion sets, pacing maneuvers from inside the “box” can result in pace-capture within the posterior wall and 1:1 conduction to the left atrium. Therefore, ablation in the mid-posterior wall (ie, within the “box” itself) **(D)** is commonly required to achieve complete posterior wall isolation. In part, this has been postulated to be due to the presence of epicardial connections. *Abbreviations:* LAA, left atrial appendage; LIPV, left inferior pulmonary vein; LSPV, left superior pulmonary vein; RIPV, right inferior pulmonary vein; RSPV, right superior pulmonary vein.

AF represents a heterogeneous disorder with a complex pathophysiologic basis. Despite significant advances made in catheter ablation therapies during the last two decades, the response to ablation varies widely, with only modest success rates observed in those with persistent AF. Yet, pulmonary vein (PV) isolation (PVI) remains the cornerstone of AF ablation, offering roughly a 50% relative reduction in its long-term recurrence (risk ratio, 0.54; 95% confidence interval, 0.33–0.89; *P* = .0147), independent of the AF type.^1^ Although the reported 1-year success rate is generally higher—about 70%–80%—in patients with paroxysmal AF,^2^ there is a clear and progressive decline in the success rate to 50%–60% during long-term follow-up using this strategy irrespective of the energy modality.^3,4^ Furthermore, PVI by itself appears insufficient for the treatment of patients with persistent AF, with 1-year success rates of about 60%,^5^ which further decline to about 20% during long-term follow-up.^6^ Although it is conceivable that the inferior results seen with PVI alone in patients with persistent AF reflect substrate-driven AF in the setting of more advanced atrial remodeling, the addition of complex fractionated electrograms^7^ or linear ablation^8,9^ has not been shown to significantly improve these outcomes. Of these, only linear ablation strategies incorporating posterior wall ablation have suggested a potential benefit.^9^ A linear ablation strategy of “box isolation” of the posterior left atrial wall, consisting of a roof and an inferior transverse line, joining the encircled PVs was initially described by Kumagai et al.^10^ with the endpoint of complete electrical isolation of the region bound by the ablation lesion set. The rationale behind this approach was the isolation of focal and re-entrant AF drivers, which are commonly found within the posterior wall,^11^ reflecting both its common embryologic origins with the PVs and also a reduction in the conducting size of the atria. In support of this notion, a mechanistic study of phase mapping in patients with persistent AF demonstrated a reduction in both the atrial critical mass and the number of AF drivers in the atrium following a posterior “box” isolation.^12^ Along the same lines, several contemporary studies^13–20^ have illustrated clinical benefits associated with posterior wall ablation/isolation in patients with persistent AF. Nonetheless, conflicting reports exist regarding the efficacy of this approach.^21^ However, of note, two independent meta-analyses^22,23^ recently illustrated a significant benefit associated with posterior wall isolation. Perhaps more importantly, both analyses found posterior wall isolation through direct ablation/debulking to be superior to the posterior “box” approach, which was in turn associated with an increased incidence of recurrent arrhythmias.^23^ Indeed, this is not surprising, as a common challenge associated with the posterior “box” strategy lies in achieving not only acute but also durable posterior wall isolation **(Figure 1)**.^24,25^ Through a series of elegant studies,^26,27^ Worck et al. investigated this conundrum and demonstrated that the durability of posterior wall isolation using the “box” method was only 46% (as assessed by a mandatory repeat electrophysiology study at 6 months). Furthermore, the authors showed that a reinforced ablation approach to achieve durable posterior wall isolation was associated with improved long-term freedom from AF. More recently, another study^28^ demonstrated a similar benefit associated with posterior wall isolation in those with paroxysmal AF. Although no differences were observed at 1 year in this multicenter study, freedom from all atrial arrhythmias and AF was shown to be superior with PVI with concurrent posterior wall isolation as compared to PVI alone during long-term follow-up lasting >3 years. Thus, moving forward, it seems prudent to differentiate between the different posterior wall ablation techniques and methodologies when examining the outcomes of clinical studies and in practice. Currently, a multicenter, prospective, investigational device–exemption, randomized controlled trial^29^ is underway to further investigate the role for PVI with posterior wall isolation versus PVI alone in patients with persistent AF.

Another strategy that has recently gained widespread recognition and increasing adoption is the vein of Marshall ethanol ablation performed either empirically for the treatment of persistent AF or in those with peri-mitral atrial tachycardias (ATs). Indeed, the complex anatomy of the mitral isthmus often necessitates further ablations to be performed epicardially within the coronary sinus. As such, ethanol ablation in the vein of Marshall has been proposed as an adjunct method. In the Vein of Marshall Ethanol for Untreated Persistent AF (VENUS) trial,^30^ vein of Marshall ablation in patients with persistent AF was associated with increased freedom from AT/AF at 1 year, particularly in the setting of peri-mitral block.^31^ A similar benefit has also been shown in those undergoing mitral isthmus ablation, as this technique significantly reduced the number of radiofrequency applications required to achieve peri-mitral block.^32^ Another study is currently underway to evaluate a stepwise ablation approach including posterior wall and vein of Marshall ablation for the treatment of persistent AF.

## Ablation techniques and strategies for scar-mediated ventricular tachycardia

**Figure 2:**
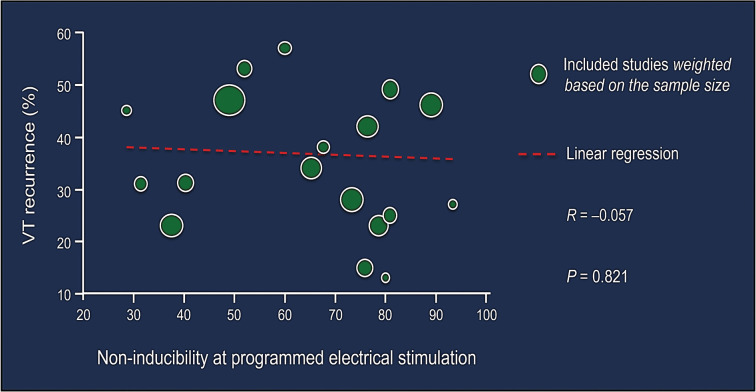
The absence of correlation between ventricular tachycardia inducibility and recurrence. Weighted meta-regression analysis assessing non-inducibility during programmed electrical stimulation at the completion of catheter ablation in predicting long-term freedom from recurrent ventricular arrhythmias in patients undergoing catheter ablation of postinfarct ventricular tachycardia. As seen, there is no discernable correlation (*R* = –0.057) between the two variables. Adapted from Santangeli et al.^35^

Similar progress has recently been made in streamlining the ablation methods for scar-mediated VT by adopting a more pragmatic approach while minimizing peri-procedural hemodynamic instability. It remains clear that hemodynamic compromise during VT ablation can severely and adversely impact the acute and long-term prognosis.^33^ In a Medicare claims analysis of 345 patients who underwent VT ablation, the authors previously showed that higher-level, intraprocedural mechanical support (using a percutaneous ventricular assist device [PVAD] vs. intra-aortic balloon pump) was associated with lower rates of in-hospital renal failure (11.7% vs. 21.7%, *P* = .01), mortality (6.5% vs. 19.1%, *P* = .001), and 30-day rehospitalization (27.0% vs. 38.7%, *P* = .04), despite a higher baseline prevalence of chronic kidney disease and heart failure in the PVAD arm.^34^ Nonetheless, the use of invasive mechanical support carries an inherent added risk of vascular injury and bleeding complications. Hence, there is a growing interest in the *“*ablation without induction” strategy.^33^ Although at first glance, this might seem counterintuitive as non-inducibility by programmed electrical stimulation is regarded as the classic endpoint for VT ablation and the only one currently endorsed by the practice guidelines, it should be emphasized that the bulk of evidence supporting non-inducibility as the procedural endpoint is derived from earlier studies of postinfarct VT. More recent studies including larger patient populations with broader disease states have offered mixed results, as reported by Santangeli et al.^35^ **(Figure 2)**. In fact, the precedent for effective, invasive VT management in the absence of VT mapping/induction is not at all a new concept. Sosa et al.^36^ previously demonstrated a 5-year VT recurrence rate of about 12% in patients with sustained postinfarct VT who underwent surgical left ventricular reconstruction that included aneurysmectomy and septal plication in the absence of intraoperative VT mapping/induction. Meanwhile, streamlining the strategy of VT ablation may encourage operators to pursue this approach not only more frequently but also as an earlier intervention.

Two decades after its publication by Reddy et al., the Substrate Mapping and Ablation in Sinus Rhythm to Halt Ventricular Tachycardia (SMASH-VT) trial^37^—an elegant and randomized trial of “prophylactic” substrate-based VT ablation shown to reduce the incidence of defibrillator therapies in patients with myocardial infarction—has failed to significantly impact or permeate clinical practice. Though the reasons for this may be complex, it seems that a more “simplified” approach coupled with the existing paradigm shift toward substrate-based mapping and ablation has once again renewed interest in VT ablation as an “early” or first-line therapy. The Effect of Early Vasopressin vs. Norepinephrine on Kidney Failure in Patients with Septic Shock (VANISH) trial^38^ recently randomized patients with previous myocardial infarction and VT to either escalated anti-arrhythmic therapy or catheter ablation. The results were overall favorable and showed a reduction in treated VT episodes, appropriate therapies, and VT burden among patients treated with ablation, even though the benefit was limited primarily to those with amiodarone-refractory VT. Similar results were reported by the Substrate Ablation vs Anti-arrhythmic Drug Therapy for Symptomatic Ventricular Tachycardia (SURVIVE-VT)^39^ trial, which randomized patients with ischemic cardiomyopathy and appropriate defibrillator shocks to drug therapy versus catheter ablation and demonstrated that catheter ablation was associated with a reduction in the composite endpoint of cardiovascular death, defibrillator shocks, and hospitalization for heart failure as compared to anti-arrhythmic drug therapy. Likewise, in the randomized Pan-Asia United States PrEvention of Sudden Cardiac Death Catheter Ablation Trial (PAUSE-SCD),^40^ early catheter ablation performed at the time of implantation of a defibrillator was shown to significantly reduce the composite primary outcome of VT recurrence, cardiovascular hospitalization, or death among patients with cardiomyopathy of various causes. Additionally, the PREVENT-VT study^41^ is currently underway to further evaluate the safety and efficacy of “prophylactic” cardiac magnetic resonance-guided ventricular substrate ablation in chronic postinfarct patients in a prospective, randomized trial.

## Novel ablation tools and energy modalities

As our understanding of the optimal techniques and strategies for ablation of cardiac arrhythmias has progressively improved, so too has our understanding of the mapping and ablation tools and technologies. In recent years, a focus has been placed on the biophysics of ablation as the advances in catheter and ablation technologies have centered on improving ablation lesion durability. In an attempt to enhance not only the procedural efficacy but also safety, many operators are increasingly monitoring the system current during energy delivery. Along with contact force, current density (ie, the ratio of the total current to the effective surface area) is a major determinant of tissue heating and lesion formation.^42,43^ Though still a surrogate, it is the best marker available. Current is to energy delivery what force-sensing is to catheter–tissue contact. While most operators aim for a target current in the range of 600–720 mA depending on the type of catheter used (eg, TactiCath [Abbott, Chicago, IL, USA] vs. ThermoCool [Biosense Webster, Irvine, CA, USA]), we need to keep in mind that current density also varies as a function of electrode size (eg, the electrode surface area of TactiCath is ∼20% smaller than that of ThermoCool, which is ∼8% larger than that of TactiFlex [Abbott]). Furthermore, there is no “safe” current cutoff/value either due to variations in the catheter tip–tissue interface (eg, a scenario in which the electrode tip is deeply embedded into the tissue). Instead, to allow increased power delivery while maintaining a low risk of tissue overheating, the current should be delivered over a larger surface area to keep its density low.

**Figure 3:**
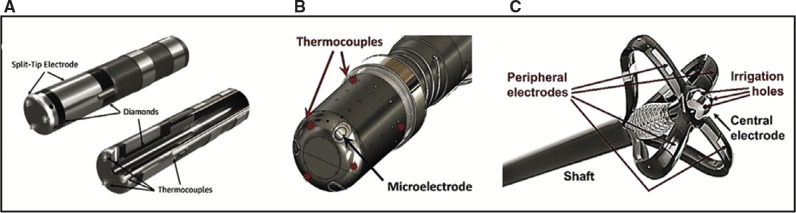
Temperature-controlled radiofrequency (RF) ablation catheters. **A:** DiamondTemp is a 7.5-French (Fr) externally irrigated ablation catheter designed to deliver RF energy via a 4.1-mm catheter-tip electrode. The tip consists of a composite electrode and two ring electrodes, both made of platinum–iridium. However, unique to the catheter’s design, the two-part composite ablation electrode tip is embedded with two industrial-grade diamonds interconnected at the distal electrode, which allow rapid heat shunting by virtue of their high thermal “diffusivity.” This permits accurate temperature estimation along the entire length of the electrode. There are three surface thermocouples at the distal end and three at the proximal end to monitor the tip–tissue interface temperature during irrigated RF ablation. In addition, the tip is electrically insulated to allow for high-resolution electrogram sensing. **B:** The 8-Fr, contact force–enabled, 3.5-mm-tip, QDOT catheter-tip design is shown, highlighting the microelectrodes and the six thermocouples. The biophysical parameters of the catheter include a 2-s pre-cooling phase, followed by a 4-s high-power (90 W), short-duration RF ablation. **C:** The 9-Fr, open-irrigated SMT (Sirona Medical Technologies) RF ablation catheter consists of a central electrode measuring 2.3 × 4 mm that is surrounded by four peripheral electrodes. The insulated-tip design is intended to block the path of RF energy into the bloodstream and the surrounding tissue in order to focus a greater magnitude of the delivered energy into the targeted tissue.

The recently developed, 9-mm sphere, lattice-tip ablation catheter (Affera, Medtronic, Minneapolis, MN, USA) has roughly a 10-fold larger effective surface area than a conventional radiofrequency ablation (RFA) catheter electrode, allowing for an energy delivery up to 600 W and 2700 mA in current. Aside from this novel design, we have also seen the emergence of other ablation systems **(Figure 3)** designed specifically for temperature-controlled RFA. Arguably, this ablation mode represents the optimal approach for targeting certain anatomical sites, such as the cavotricuspid isthmus, given its broad anatomical variations, which can sometimes increase the likelihood of non-homogenous tissue heating, thereby leading to a steam pop. Furthermore, high-power, short-duration, temperature-controlled RFA has the potential to improve the procedural efficiency by shortening the ablation and procedure times. Additionally, by minimizing conductive heating and augmenting resistive heating during energy delivery to the left atrial wall, this may have the potential to reduce collateral injury, but this still remains to be determined. In the swine model, high-power, short-duration RFA at 90 W resulted in more contiguous lesions than conventional ablation with a wider diameter but shallower depth.^44^ Clinically, two independent analyses, the single-center Prospective Randomized Evaluation of High Power During CLOSE-guided Pulmonary Vein Isolation (POWER-AF) and the Clinical Study for Safety and Acute Performance Evaluation of the THERMOCOOL SMARTTOUCH SF-5D System Used with Fast Ablation Mode in Treatment of Patients with Paroxysmal Atrial Fibrillation (QDOT-FAST) multicenter trial, have also illustrated the feasibility and the procedural efficiency associated with high-power, short-duration RFA in patients with paroxysmal AF.^45,46^ Nonetheless, both studies still reported esophageal complications, including a hemorrhagic esophageal ulcer in one and an esophageal ulcerative perforation in the other, possibly suggesting a narrower margin of safety associated with this approach when applied to the posterior wall than previously thought.

Another novel RFA catheter design is the “ultra-efficient” insulated-tip SMT catheter (Sirona Medical Technologies, Windsor, CT, USA), which is capable of diverting a greater magnitude of the delivered energy into the targeted tissue **(Figure 3C)**. This catheter was specifically designed to minimize the passage of energy into the bloodstream and the surrounding tissue.^47^ This is achieved by insulating the central electrode, which is surrounded by an insulating dielectric and a flexible metallic braid that acts as a Faraday cage. Unlike conventional RFA catheters, with which ∼90% of the delivered energy dissipates either into the bloodstream or the surrounding tissue, the SMT allows for delivery of ∼67% of the delivered energy (radiofrequency/pulsed field) directly into the targeted tissue.^47^ Accordingly, low-power, low-irrigation (8–12 W, 2 mL/min) RFA using this catheter proved capable of creating more uniform but comparable-sized lesions to those of a conventional, 3.5-mm-tip RFA catheter (30 W, 30 mL/min) with a lower incidence of charring, steam pop, or tissue cavitation.^47^ On the contrary, high-power, high-irrigation (15 W, 20 mL/min) RFA using the SMT catheter yielded lesions that were much larger and deeper than those achieved with the conventional RFA catheter.

**Figure 4:**
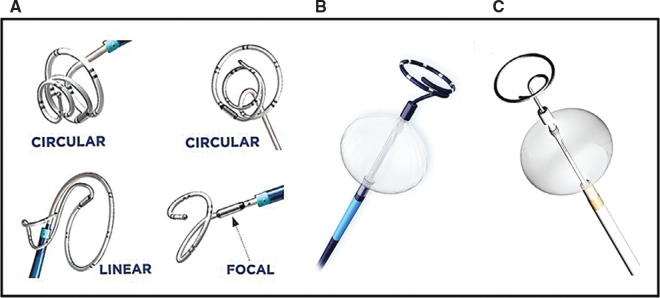
Novel cryoablation catheters. **A:** The Adagio Medical catheter uses a high-pressure “near-critical” nitrogen refrigerant near its boiling temperature of −196°C to achieve ultra-low temperatures. The distal section of the catheter is flexible and contains 12 electrodes in the cryoablation element and eight electrodes in the diagnostic section. As depicted, the cryoablation element and diagnostic section are shaped in vivo by endoluminal stylets to create circular, linear, or focal lesions. **B:** The design of the POLARx cryoablation system (Boston Scientific, Marlborough, MA, USA) is an 11.8-French catheter with a lumen able to accept a 0.035″ guidewire made up of a compliant thermoplastic material that expands to 28 mm in diameter when inflated to 2.5 psi and 31 mm when inflated to 7.5 psi. **C:** The design of the Synaptic cryoballoon (Synaptic Medical Corp., Carlsbad, CA, USA) is similar to the latter, as it is a 12-French over-the-guidewire/mapping catheter balloon. The distal end consists of a compliant polyurethane balloon with a diameter of 28 mm when inflated to 3 psi and 31 mm when inflated to 5 psi. The shafts of both catheters contain injection tubes that deliver liquid nitrous oxide as a source of coolant into the balloon.

Similar to the recent advances made in the RFA space, improvements have also been made to cryoablation catheters. Not only have compliant and expandable cryoballoons been introduced that are capable of expanding to 31 mm in diameter, providing 20% greater ablation surface areas **(Figure 4)**, but newer and more innovative catheters capable of ultra–low-temperature cryoablation have also emerged. Until recently, cryoablation using nitrous oxide as cryogen (boiling temperature, −89°C) was the only endovascular modality available in clinical practice. More powerful cryoenergy systems have been primarily used during open surgical ablations but cannot be readily translated to percutaneous ablation platforms due to technical challenges in coolant delivery. Ultra–low-temperature cryoablation can overcome the said challenges and substantially increase the cryogenic power available for transcatheter ablations using a high-pressure “near-critical” nitrogen refrigerant (near its boiling temperature of −196°C).^48^ In animal models, through the use of a novel cryoablation system (Adagio Medical, Laguna Hills, CA, USA) **(Figure 4)**, this has been shown to reliably produce durable, transmural lesions not only in the atria but also in the ventricle.^49^

**Figure 5:**
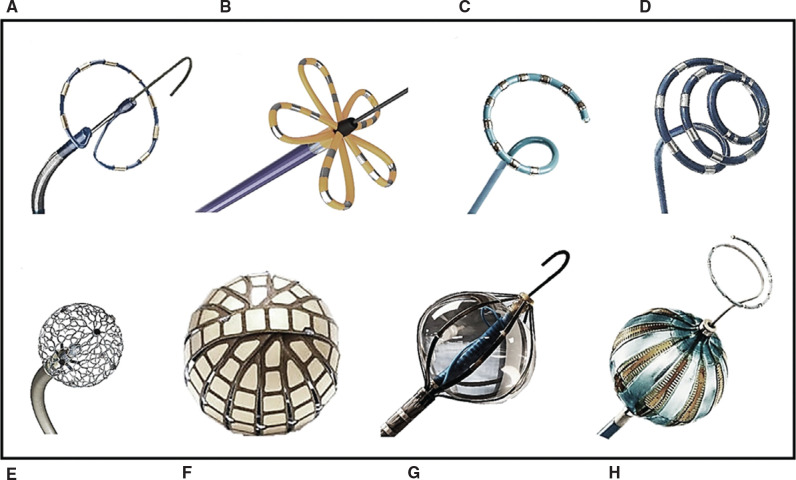
Novel and emerging pulsed field ablation (PFA) catheters. **A:** The PulseSelect ablation catheter (Medtronic) is a 9-French (Fr), 25-mm, nine-electrode, biphasic, bipolar circular array catheter (electrode length, 3 mm). **B:** Farawave (Farapulse, Menlo Park, CA, USA) is a 12-Fr, over-the-guidewire PFA catheter that consists of five splines, with each spline carrying four electrodes, available in two maximal diameter sizes of 31 and 35 mm. The outermost electrodes may be used for electrogram recording and pacing. **C:** VariPulse (Biosense Webster) is an 8.5-Fr, multi-electrode, irrigated (4 mL/min), steerable, variable-loop circular ablation catheter. The 10 platinum/iridium electrode rings are used for visualization, stimulation, recording, and bipolar PFA. All 10 poles are used for ablation, except in the case of electrode overlap, which would require the most distal and proximal electrodes to be disabled. **D:** The ElePulse PFA catheter (CRC EP, Tustin, CA, USA) design consists of an 8-Fr, 16-electrode, bidirectional, spiral mapping/ablation catheter intended for single-shot ablation. The catheters are available in two sizes (25 and 30 mm) with radiopaque tips, inserted through a standard 8.5-Fr introducer. The larger 30-mm catheter has four distal mapping electrodes. The PFA system delivers microsecond-wide, QRS-gated, bipolar, biphasic pulsed fields, allowing for ablation using all or individually selected electrodes. **E:** Sphere-9 (Affera, Medtronic) is a lattice-tip catheter with a 10-fold larger effective area than a conventional 3.5-mm electrode that is able to toggle between temperature-controlled radiofrequency ablation and PFA. As such, it is capable of delivering much greater energy with a lower risk of tissue overheating. The catheter uses an expandable, 9-mm spherical, nitinol tip that serves dual functions of mapping and ablation. Its 7.5-Fr shaft is bidirectionally deflectable. Nine mini-electrodes/thermocouples are distributed around the expandable electrode to provide electrogram/temperature feedback. **F:** The Globe (Kardium, Burnaby, BC, Canada) consists of a 30-mm array comprised of 16 ribs with a total of 122 electrodes capable of mapping, pacing, and assessing tissue contact and temperature while delivering radiofrequency or pulsed-field energy. Each rib has between seven and nine electrodes, the sizes of which range from 9–13 mm^2^. The interelectrode distance along the ribs is only 0.8 mm with 1.3–1.8 mm between the ribs. The catheter must be inserted through a specifically designed 19-Fr deflectable sheath. **G:** Volt (Abbott) is a 12.5-Fr, 28-mm, eight-spline, bidirectional, variable-diameter, over-the-guidewire, magnetic sensor-enabled balloon PFA catheter currently awaiting a first-in-human evaluation. **H:** A proposed PFA balloon design (Biosense Webster) that consists of a 28-mm, 10-electrode, magnetic sensor-enabled PFA catheter equipped with an inner-lumen circular mapping catheter.

**Figure 6:**
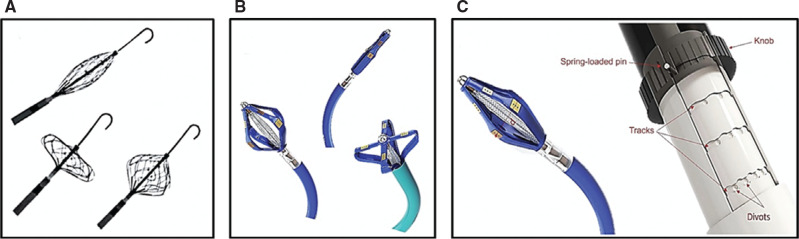
Emerging pulsed field ablation (PFA) catheter systems with variable/multimodal configurations. **A:** A novel, 8-French, variable-configuration, single-shot PFA catheter (SpherePVI; Affera, Medtronic) that has a large, compressible/conformable lattice framework that can be used and deployed in an oval, a spherical, or a flat configuration capable of expanding up to 34 mm in diameter. The catheter consists of six sections that are independently and sequentially energized for ablation. **B:** The multimodal SMT catheter (Sirona Medical Technologies) that can be deployed in focal, oval/circular, or flat configurations with an axial rotating mechanism that allows the distal array to rotate about the pulmonary veins to achieve contiguous, circumferential radiofrequency/pulsed-field lesions to eliminate the need for catheter repositioning. **C:** A close-up of the multimodal SMT array’s rotating mechanism. Moving the knob along the handle axis changes the array’s diameter (d) to adjust to the pulmonary vein diameter. The same knob is also used to axially rotate the array. As the diameter of the array increases, the angular rotation becomes smaller to create the same linear movement in the ablation electrodes. This adjustment is achieved using the tracks on the handle. The spring-loaded pin engages with the divots as it clicks in to limit the extent of angular rotation (the larger the array diameter, the shorter the spacing between the divots). The curved track allows the array to close slightly, lifting the array off the tissue to prevent excessive friction with the tissue while rotating the mechanism.

Yet, the most disruptive of the recent developments in cardiac electrophysiology is that of pulsed-field ablation (PFA), a primarily non-thermal ablative strategy that achieves cell death via electroporation. PFA utilizes intermittent, high-intensity electric fields for short durations (micro- vs. nanoseconds) to achieve irreversible cellular and tissue destruction.^50^ Its potential advantage over thermal ablation lies in the preferential targeting of myocardial tissue with a reduction in collateral damage to the esophagus or the phrenic nerve, as demonstrated in prior preclinical studies.^51,52^ Although, in a recent study, intentional PFA of a deviated porcine esophagus from within the adjacent inferior vena cava did reveal acute non-thermal, PFA-related histological changes in the muscularis layer of the esophagus, these changes were transient and completely resolved by 21 ± 5 days of follow-up.^53^ Presently, a variety of different PFA catheter designs and systems with single- **(Figure 5)** or multimodal **(Figure 6)** configurations are being introduced, some of which are capable of combined PFA and RFA. In addition, the role of PFA for ventricular tissue ablation is also under investigation.^54–56^ Nonetheless, unresolved issues surrounding PFA remain. These include efficacy—namely, acute/intraprocedural assessment of reversible versus irreversible electroporation—as well as safety concerns largely related to electrolysis giving rise to microbubbles within the blood pool with the potential for micro-emboli, hemolysis, and hemoglobinuria with resultant acute kidney injury and also severe coronary spasm.^57,58^ A recent computer modeling study^59^ of the variety of different catheter designs suggested greater efficacy with balloon technologies followed by flexible polymer splined and circular catheters. At similar settings, circular catheters were found to have four times less efficacy than balloon/flexible splined catheters due to greater electrode exposure to the blood pool, which resulted in current shunting. With respect to energy delivery, a multi-unipolar strategy (ie, all electrodes energized positively with simultaneous current flow from all electrodes to the back electrode) emerged as the most efficacious method. Having said that, the electroporation risk to the aorta was highest with such an approach, whereas the microbubbling risk was deemed lowest with a balloon-based catheter.

## Catheter ablation in the ambulatory setting

Though further research and investigation into the various PFA technologies are warranted, it seems that the next few years will bring remarkable and innovative technologies to the field. These developments will most certainly improve catheter ablation procedures and further enhance patient and procedural outcomes. Meanwhile, a question that has come up repeatedly is whether these procedures could be performed safely and effectively in the outpatient ambulatory setting. In many regards, the periprocedural management of patients who receive catheter ablation is similar to those undergoing ambulatory surgery. Given the safety of same-day discharge of patients following arrhythmia ablations (eg, AF), there has been a growing interest in expanding catheter ablations to a completely ambulatory setting. Such an approach would have the potential to yield not only higher efficiency but also considerably greater savings for the health care system. Zagrodzky et al.^60^ reported on the feasibility and safety of cryoballoon ablation of AF in a small cohort of patients in a free-standing ambulatory center. While procedural safety, efficacy, and durations were all similar to those in the hospital, the length of stay was significantly shorter in the ambulatory versus hospital setting (5.4 ± 1.5 vs. 13.0 ± 13.0 h, *P* < .01). More recently, Willcox et al.^61^ reported on the outcomes of 476 patients who underwent AF ablation (85% using cryoballoon) in the ambulatory setting over a 6-year period. Once again, the study found AF ablation to be safe and feasible in the ambulatory setting, with a 13.7% rate of hospital/emergency department visits within 30 days, which is essentially similar to that reported for same-day AF ablation discharges performed in the hospital setting. Moreover, the authors concluded that, with the availability of a 23-h ambulatory stay, ultrasound, and computed tomography, all complications encountered in the study could have been successfully managed without hospital/emergency department interventions. It is important to recognize that the significantly shorter lengths of stay and lower costs to the health care system associated with catheter ablations performed in an ambulatory setting could not only yield marked reductions in resource utilization and increased cost savings but also greatly improve global access for patients in need of catheter ablation therapies. Though many believe that the time for a shift in cardiac electrophysiology procedures to the ambulatory setting has already arrived, without payer-approved reimbursement (namely—the Centers for Medicare & Medicaid Services), such practices remain small and limited to only a handful of facilities. Nonetheless, and despite the outcomes of a payment ruling, with so much lying ahead and on the horizon, the future of cardiac electrophysiology remains nothing short of bright and exciting.
